# A geospatial hybrid platform to support public policy-making and monitoring for community-based food management and security in the context of global climate change: A study protocol

**DOI:** 10.1371/journal.pone.0342334

**Published:** 2026-03-10

**Authors:** Carlos Matías Scavuzzo, Micaela Natalia Campero, Fernando Roda, Carlos Marcelo Scavuzzo, María Daniela Defagó

**Affiliations:** 1 Instituto de Altos Estudios Espaciales Mario Gulich, Universidad Nacional de Córdoba, Comisión Nacional de Actividades Espaciales, Falda del Cañete, Córdoba, Argentina; 2 Centro de Investigaciones en Nutrición Humana, Escuela de Nutrición, Facultad de Ciencias Médicas, UNC, Argentina; 3 Consejo Nacional de Actividades Científicas y Técnicas, Córdoba, Argentina; 4 Escuela de Nutrición, Facultad de Ciencias Médicas, Universidad Nacional de Córdoba, Córdoba, Argentina; 5 Fundación InnovaComunidad, Córdoba, Argentina; 6 Instituto de Investigaciones en Ciencias de la Salud, Consejo Nacional de Actividades Científicas y Técnicas, Córdoba, Argentina; PLOS, UNITED KINGDOM OF GREAT BRITAIN AND NORTHERN IRELAND

## Abstract

In Latin America, Food and Nutritional Insecurity (FNI) is a challenge, particularly in households that receive social assistance programs, where food scarcity affects up to 50% of these households. Environmental degradation and climate change are significant contributors to FNI, underscoring the importance of ongoing monitoring. In this paper, we present the roadmap for a higher-impact project to support Food and Nutrition Security (FNS) policymaking in Latin America. The ultimate goal is to improve FNS in communities affected by climate change through the development of an interactive platform that evidences the identification of variables, a transnational data acquisition program, the development of predictive models, and the assessment of climate vulnerability in the region. Additionally, an open data platform, together with the dashboard and a virtual assistant, is being developed for monitoring FNS indicators in Latin America. The project was awarded the ALSEA prize and addresses technological and regional challenges through a multidisciplinary and international team. Effective coordination between space agencies, academia, government, and the productive sector is required to ensure that project results are usable and add value at the local level. The Supporting Evidence for the Proposed Approach presented in this paper are promising and pave the way for future developments that extend not only the geographic scope but also the dimensional analysis.

## Introduction

Food and Nutrition Security (FNS) is defined as the condition in which all people, at all times, have physical, social, and economic access to sufficient, safe, and nutritious food to meet their dietary needs and food preferences for an active and healthy life [1]. Despite global and regional progress, Food and Nutritional Insecurity (FNI) continues to affect large segments of the population in Latin America. In 2022, an estimated 43.2 million people in the region experienced hunger, and 247.8 million were affected by moderate or severe FNI, which entails a reduction in the quality or quantity of food consumed [2].

This crisis is deeply rooted in the interaction between structural socioeconomic inequalities, environmental degradation, and political instability. Climate change has emerged as one of the key drivers of FNS challenges in the region, affecting agricultural productivity, water availability, and the resilience of local food systems [3]. From the One Health perspective, the disruption of ecological, animal, and human health interfaces further aggravates these impacts [4]. As a result, FNI is linked to a range of negative outcomes, including child undernutrition, impaired physical and cognitive development, decreased labor productivity, and increased vulnerability to disease [2,5]. Furthermore, the region suffers from insufficient and unstable public policy frameworks that limit long-term adaptation and response capacity to both environmental and social shocks [1].

Although the Plan for Food Security, Nutrition and Hunger Eradication promoted by the Community of Latin American and Caribbean States (CELAC) is a key regional initiative encouraging local food production systems, current strategies fall short in integrating the multidimensional nature of FNS and its dynamic interaction with climate variability [6]. Particularly lacking are tools and information systems that allow for continuous, spatially-explicit monitoring and modeling of FNS indicators to support evidence-based policy design, implementation, and evaluation [7,8].

The ALSEA Award is an international award promoted by ALSEA Foundation in alliance with World Vision, aimed at supporting innovative scientific projects that advance food and nutrition security in Ibero-America. The prize grants up to USD 150,000 to initiatives aligned with the Sustainable Development Goals (SDGs), especially SDG 2 (Zero Hunger) and SDG 3 (Good Health and Well-being). It is open to research teams from Argentina, Chile, Colombia, Mexico, Paraguay, Spain, and Uruguay, and prioritizes proposals that are interdisciplinary, evidence-based, and capable of informing public policy. The selection process includes a competitive peer-reviewed evaluation conducted by an international panel of experts to ensure scientific rigor, relevance, and social impact [9].

In response to the need outlined above, this article presents a novel, award-winning (ALSEA) project aimed at developing an integrated simulation ecosystem to assist decision-making in the context of FNS and climate change in Latin America. The ecosystem combines artificial intelligence (AI), geospatial technologies, and open-access data to monitor, model, and predict the behavior of Community Food Environments (CFEs). The initiative is beginning to be implemented in six countries: Argentina (AR), Chile (CH), Uruguay (UR), Paraguay (PY), Peru (PE), and Colombia (CO), with the intention of advancing in the future over other countries in Latin America and the Caribbean. Its central objective is to provide governments and stakeholders with an interactive, evidence-informed platform to simulate future scenarios (10, 30, and 50 years ahead) and evaluate the potential impacts of climate change and public policy interventions on FNS outcomes.

The project is structured into six interconnected work packages (WP), encompassing data architecture, predictive modeling, scenario simulation, real-time data integration, science communication, and project management. Its main innovation lies in leveraging open-access satellite imagery and sociodemographic data to produce low-cost, scalable tools for FNS monitoring and policy support. The system integrates machine learning and geospatial analysis to identify vulnerable areas, simulate climate and policy scenarios, and assist decision-making through an interactive dashboard and a generative AI-powered virtual assistant.

This transdisciplinary approach not only bridges the gap between high-level data analysis and on-the-ground decision-making but also addresses the persistent “last mile” problem of translating scientific insights into actionable policies. By combining expertise in nutrition, epidemiology, data science, geospatial analysis, and public health, the project offers a flexible, regionally-adaptable solution aligned with SDG 2, SDG 3, SDG 6 (Clean Water and Sanitation), SDG 10 (Reduced Inequality), and SDG 13 (Climate Action).

## Materials and methods

The project aims to support the design of public policies to enhance FNS in communities impacted by climate change, leveraging machine learning over geospatial data analysis. This challenge is addressed through the following specific goals:

- Define dimensions of analysis, variables, and FNS indicators.
- Identify Data Sources for the participating countries and design a Data integration Strategy.
- Develop predictive models to simulate future scenarios on the basis of climate change.
- Develop an interactive dashboard for FNS monitoring that highlights the key areas of public policy making.
- Design an application interface for real-time field data feeds using mobile devices.
- Implement a natural language processing-based virtual assistant to facilitate FNS insights discovery.

The project is designed to support policymakers with advanced decision-making tools. Thus, we can differentiate between direct users and Indirect beneficiaries. First ones refer to the main target group is decision-makers, including public officials and policymakers in the field of food security and other dimensions of human security. These users will employ the integrated Dashboard and API ecosystem to design, plan, execute, monitor, and adjust evidence-based public policies. Using this system will enable them to perform accurate simulations, reducing the likelihood of errors in the implementation of specific strategies.

More broadly, the final target population includes all inhabitants of the study regions, as this platform is an educational tool with impact. The positive results derived from the use of this system by decision-makers will impact the quality of life and food security of the entire population. Thus, although indirectly, the general population will benefit from more effective public policies that are adapted to the changing conditions imposed by climate change.

### Study design

Methods involve open data sources, regaining resources previously invested in data collection. Features linked to CFEs were brought to urban localities with 5,000 inhabitants or more for each participating country (AR-UR-PY-PE-CH-CO). Open data (including political, sociodemographic, and specific variables on health indicators) will be searched for from various official open secondary and tertiary sources using techniques such as web scraping and web crawling. However, it is well known that when integrating heterogeneous data sources, disparities in data types and formats, and ambiguity present significant interoperability barriers, along with data quality issues.

That is why a strategic approach was proposed to standardize and harmonize data to support the construction of interoperable indicators that enable FNS surveillance and prediction. Likewise, the aim is to develop a data control plan that ensures quality and consistency through data validation and transformation. Different scales and years are used in data collection (but the main focus will be on the locality scale), carefully related to each other to maintain conceptual robustness in the predictions. Given the minimum unit of analysis for each database, data processing blocks will be executed at scales. At last, databases will be assembled using merge, join, and attribute

Open sociodemographic, economic, and public policy data are processed, such as multidimensional poverty, health coverage, food assistance, and other variables included in the attached documentation. Environmental and climate data will be extracted from open portals such as Worldclim, ESA-Copernicus, USGS, and Google Earth Engine. Medium- and high-resolution satellite images from missions such as Sentinel 2, Landsat 8, MODIS, among other products, were processed. Environmental indices, air and water quality indicators, heat waves, precipitation, etc., are calculated. Also, spatial statistics will be used to assess randomness and detect spatial clusters (Moran's Index, LISA, Optimized Hot Spot Analysis, multivariate clustering), and thematic mapping of all variables will then be generated to obtain reports from the databases.

Machine learning solutions are developed based on models that have proven to deliver excellent results in this type of task. Drawing on the team's experience, a development process will be carried out involving everything from the definition and implementation of metrics to be optimized that reflect the problem to be modeled, feature engineering techniques such as Shap-Values, and exploratory data analysis to select the set of variables that best model the problem, and model selection through techniques such as cross-validation for each country. This modeling is the core of the solution that is used to perform simulations and evaluate various scenarios, such as climate change and possible public policy interventions.

For the visualization and interpretation of previous, new, modeled, and simulated data, we propose developing an interactive control panel using ArcGIS Dashboard, a tool that allows the creation of control panels and management dashboards that facilitate access to heat maps, graphs, lists, and summary statistics, which will be automatically updated based on user interaction. Users can interact with these dashboards to explore data in depth and obtain detailed information by navigating and selecting different layers and attributes.

Integration of data from national surveys and APIs for real-time data collection using ArcGIS GeoEvent is proposed to design. This tool enables real-time processing and analysis of geospatial data streams, allowing dynamic data to be incorporated directly into dashboards. This allows users to visualize and analyze emerging trends and make informed decisions based on the latest available information. This will improve the sensitivity of models for simulations and also allow for remote, real-time monitoring of field operations by visualizing the data entered by researchers.

Finally, a virtual assistant based on a large language model (LLM) such as GPT will be implemented, utilizing generative AI. This assistant will aim to provide evidence-based recommendations on the management of FNS and CFEs. Functionally, this assistant will engage in conversation with the user, responding to queries related to the simulations performed by the trained models.

### Work flow

The project is implemented by an international, interdisciplinary team with demonstrated expertise in open and geospatial data management, predictive modeling, and the development of interactive platforms. The plan of work is structured into six interrelated WP, each with defined objectives, tasks, and outputs as applicable:

- WP1 – Data architecture and predictive modeling in local case studies

This focuses on the adjustment and training of models for AR, CH, UR, PY, CO, and PE. The tasks include: 1) Identification and standardization of key predictive variables and development of FNS indicators; 2) Analysis of spatial patterns and associated determinants of FNS and its core pillars (access, availability, consumption, and biological utilization); 3) Geospatial characterization of CFEs, incorporating socioeconomic, environmental (e.g., water and food availability, climate), and policy dimensions; 4) Environmental assessment based on satellite data (climate, land use, vegetation, etc.); 5) Development, training, and validation of machine learning models optimized for FNS metrics; 6) Simulation of future scenarios (10, 30, and 50 years) under different climate change projections and public policy interventions.

Expected results of this WP: Vulnerability and cluster maps, technical reports, trained and validated AI models adaptable to various contexts.

- WP2 – Climate-policy simulation dashboard

This WP aims to develop an interactive, dynamic dashboard to support political decision-making, allowing users to visualize data trends, identify priority areas for intervention, and simulate alternative policy scenarios. The dashboard will be user-friendly and serve both technical staff and decision-makers.

Expected results: Pilot-tested dashboard, external validation report, technical documentation, user manual, and training workshops.

- WP3 – Real-time field data integration

This WP addresses the integration of real-time data through the development of mobile interfaces and APIs. Field data may be collected directly from individuals and communities or obtained from public national and provincial surveys. These data streams will continuously feed the models and dashboard, enhancing prediction sensitivity and timeliness.

Expected results: Pilot-tested APIs, external validation report, technical documentation, user manual, and training workshops.

- WP4 – AI-powered virtual assistant

This component focuses on the development of a virtual assistant based on natural language processing (NLP) and generative AI. The assistant will interact with users, interpret dashboard results, and execute predictive models in the background. It will provide automated responses and tailored recommendations for policymakers in real time.

Expected results: Technical report, assistant user and maintenance manuals, training workshops, and localized reports.

- WP5 – Science communication strategy

This WP is dedicated to the design and implementation of a comprehensive public science communication strategy. It will address both academic dissemination (peer-reviewed articles, conference presentations) and public engagement through mass media and digital platforms. The goal is to enhance transparency, outreach, and the societal value of the project’s outputs.

Expected results: Scientific publications, conference materials, press releases, audiovisual content (videos, podcasts), and outreach reports.

- WP6 – Project coordination and management

This final WP covers all project management activities. It ensures results-oriented coordination in a collaborative environment, emphasizing transparency, shared values, and the personal well-being of the team. Evaluation of project progress and team dynamics will be conducted through structured monitoring processes.

Expected results: Internal project calendar, dynamic task scheduling, periodic progress reports, and perception evaluations regarding team cohesion and performance.

**Fig 1:**
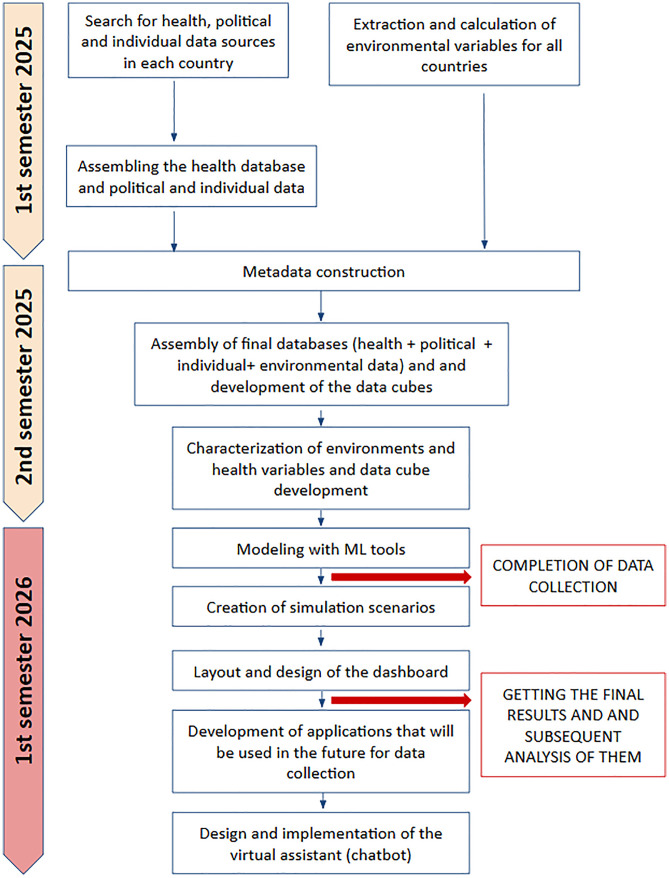
General timeline and methodology of this proposal.

In summary, Fig 1 shows the main methodological stages to be completed, including estimated dates for the completion of data collection and the obtaining of final results (which will be reflected in the proposed platform).

### Ethical considerations

This study follows an ecological design where observational units are the localities (not the individuals or households evaluated in each data source used to compile the national databases).

In all cases, the socioeconomic, political, and environmental information to be extracted mainly from national censuses was free of any sensitive data at the individual level and had the corresponding licensing enabling its use. Similarly, the health data extracted, although more sensitive in nature, did not contain any personal identification either.

In addition, the scale of all health variables in most countries, except Peru, was at a scale greater than the locality. In the case of Peru, nutritional status information was at the health institution level, so prevalences were calculated and averaged by locality.

Given that no data of any kind will be collected in this proposal, it will not be essential to submit the proposal to the relevant ethics committee(s). However, throughout the process, absolute caution was exercised regarding the quality and scale of the data. As a result, the assembled databases will be the available databases with the appropriate licensing, identifying only the city in question, enhancing the availability of information. Thus, international ethical standards such as the Declaration of Helsinki were respected throughout the data extraction and data processing flow.

### Supporting evidence

Although the project is still in its early stages, some significant supporting evidence and background concepts for the Proposed Approach, particularly in relation to WP1 and WP2, are presented in the following sections:

Operationalization of Predictive Variables and Food and Nutrition Security (FNS) Indicators.

The initial phase of this project focuses on identifying the predictive variables and health outcome indicators (dependent variables) to be employed in monitoring FNS and CFEs, as well as in the development of predictive models for future scenario simulation. The finest spatial resolution adopted for analysis is the city.

The specific set of variables to be incorporated into each predictive model is contingent upon the availability and accessibility of relevant data in each participating country. Consequently, both the configuration of predictors and the model specifications will be customized to reflect the data ecosystem of each national context. Nevertheless, the proposed modeling framework is structured around theoretically grounded relationships among classes of variables, which must be substantiated with empirical data to ensure the validity and robustness of the predictions.

The independent variables—hereafter referred to as contextual variables—capture a range of socioeconomic, demographic, political, and environmental characteristics at the locality level. These include indicators such as population distribution by sex/gender and age, health insurance coverage, poverty rates, levels of unmet basic needs, educational attainment, employment status, access to social protection programs (particularly food-related), household income, and degrees of social exclusion. In parallel, the dependent variables represent population health outcomes, particularly nutritional status among children and adolescents (ages 0–17), encompassing various forms of malnutrition, including low birth weight, macrosomia, emaciation, overweight, and obesity.

Additionally, a subset of variables reflects the physical/climatic environment in which food systems operate. These environmental variables describe the biophysical conditions that influence the functionality and resilience of food supply chains.

Thus, if equivalent datasets can be obtained across all participating countries—replicating the structure already available for Argentina—the contextual variable set includes approximately 30 policy-related indicators, 30 demographic indicators, and 30 environmental indicators. These are complemented by 15 health-related dependent variables, enabling harmonized modeling across national contexts.

To facilitate cross-country modeling and scenario analysis, a harmonized multi-dimensional data cube will be constructed for each country, integrating all contextual and outcome variables within a geospatially referenced framework.

**Fig 2:**
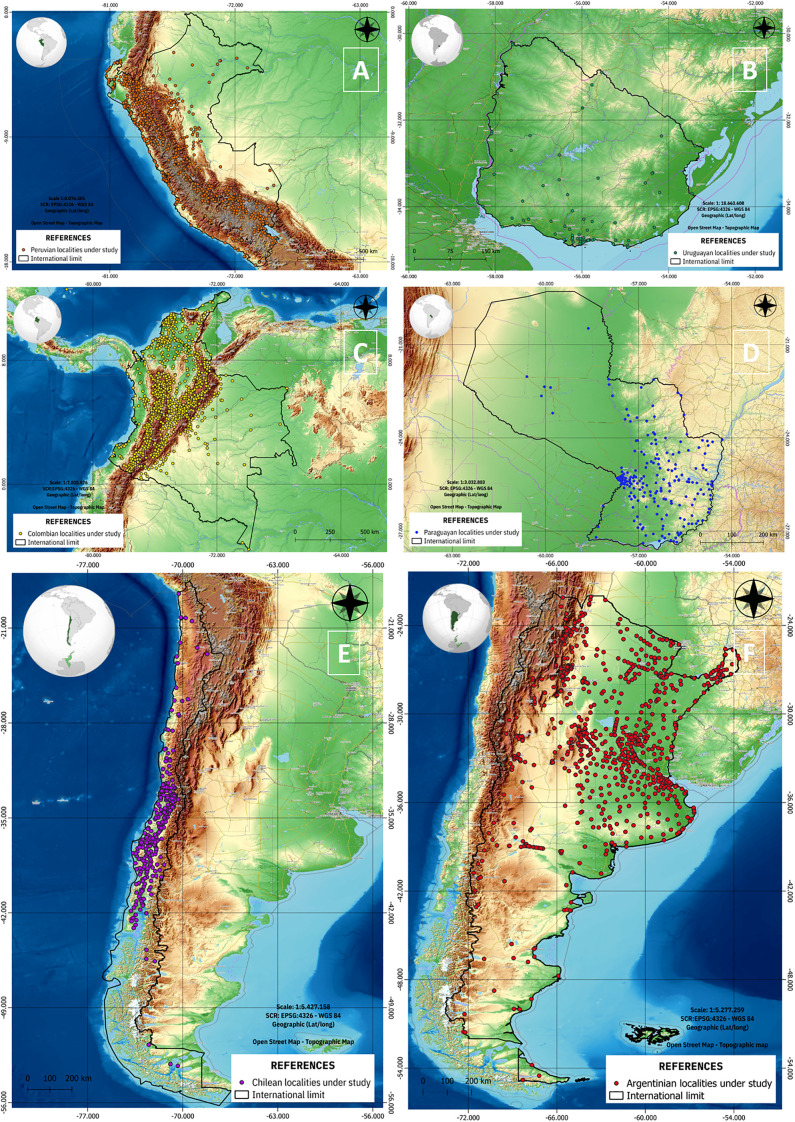
Locations included in the project by country.

**Fig 3:**
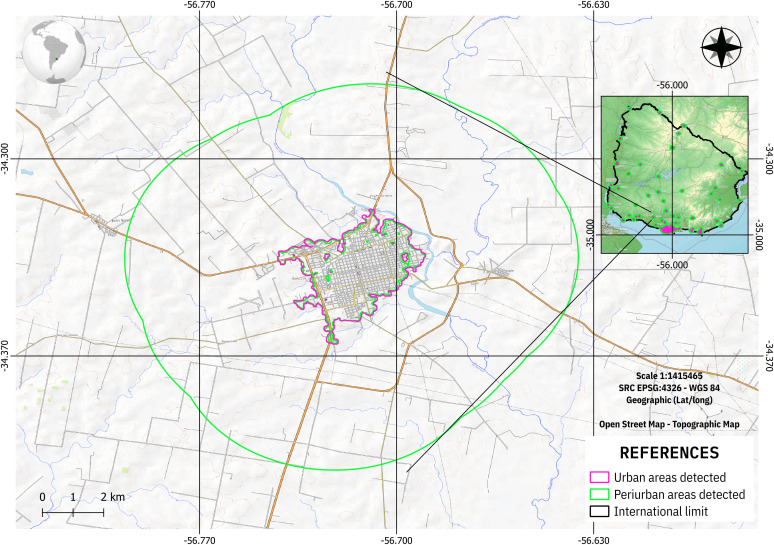
Urban and periurban areas identified.

In Fig 2, it can be seen the 3.072 localities included in the project so far for the 6 countries: 721 in Peru (map A), 76 in Uruguay (map B), 921 in Colombia (map C), 222 in Paraguay (map D), 322 in Chile (map E), and 810 in Argentina (map F). An important spatial consideration is the extraction of environmental variables through the application of two concentric spatial buffers around each city. The first buffer delineates the urban core, while the second captures a nearly 5 km peri-urban area. Fig 3 illustrates an example of these buffer zones for one selected city in Uruguay.

### System architecture

**Fig 4:**
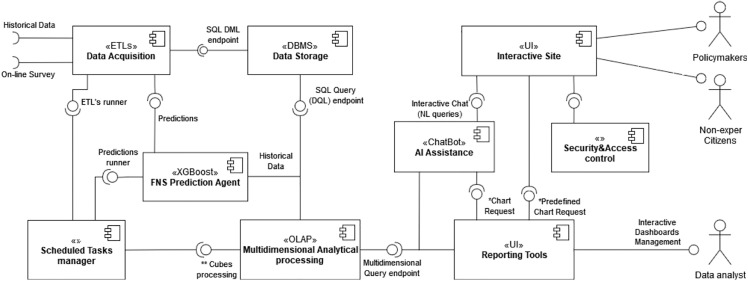
System Architecture proposed.

The project's various workflows address the development of key components of a data workflow, encompassing everything from data acquisition, curation, and processing to its publication and integration into decision-making processes. This workflow is the responsibility of an information system that must be developed to organically integrate each of these components through a modular design that facilitates project maintenance and scalability. Fig 4 shows the proposed system architecture using a component diagram (using UML notation).

The planned components for the system are:

- Data Acquisition: This module is responsible for connecting to the different data sources used and carrying out the Extraction, Transformation, and Load (ETLs) processes for loading data into local repositories. For data extraction, this module must be able to access three types of interfaces: i) Repositories with historical data from each country (social, political, environmental, health, etc.); ii) Online surveys with updated information collected in the field by a network of collaborators using a multiplatform form system; iii) On-demand predictions about health variables carried out by an intelligent agent (i.e., FNS Prediction Agent). The transformation processes are responsible not only for data cleansing, curation, disambiguation, and normalization tasks, but also for managing the associated metadata. The module uses the database manager to store the resulting data in the system's database.
- Data Storage: It is a classic DataBase Management System (DBMS). A Relational DataBase is considered in this proposal. The DBMS must provide SQL endpoints (for data access and update) as well as data integrity and security services.
- FNS Prediction Agent: This module implements a model-based intelligent agent as described in the section below “Preliminary Modeling of Health Variables Under Climate Change Scenarios: The Case of Argentina” (XGBoost-based) as an ML Agent. During training, the agent queries the DB for historical data for a requested spatio-temporal region. On the other hand, the agent provides an on-demand prediction service for health variables based on socio-political-environmental scenarios using the trained models. While this agent will be able to respond online to queries launched directly by the end user, in this first version of the system, the agent periodically provides the Data Acquisition module with predictions for a predefined set of scenarios (i.e., predictor patterns). The periodicity of this process, as well as the model training routines with new data, are triggered by the Scheduled Task Manager.
- Scheduled Task Manager: Responsible for executing the system's scheduled tasks. These include ETL execution, cube processing, and agent training with newly acquired historical data.
- Multidimensional Analytical Processing (MAP): While the data is stored in tabular format in the Relational DB, this module is responsible for providing multidimensional views of the data. In other words, it is responsible for OLAP (Online Analytical Processing) services for defining and maintaining cubes, dimensions, measures, and granularity levels, aggregation functions, etc. This module has two key functions: i) processing cubes by calculating and storing aggregations offline to balance the processing load at query time; ii) providing query services to the cubes with operations such as filtering, drill-down, roll-up, slicing, etc.
- AI Assistance: Implements an LLM-based chatbot primarily intended to assist non-expert users in processing SAN-EAC data. These users can access this service through an interactive web page (interactive site component). To answer queries, this assistant must be able to retrieve data by querying cubes using the multidimensional data endpoint. Furthermore, the agent is expected to request charts and dashboards from the reporting service to help this type of user obtain graphical representations of the data. The first experiments with this assistant are presented in the section below, “Chat or DASH Prototype”.
- Reporting Tools: The system incorporates a reporting service to provide on-demand geospatial-based dashboards and dynamic reports. By interacting with the MAP component, this service builds interactive views of the stored data cubes on the fly. Two main clients interact with this component: I) Data Analysts who can create, manage, and publish new reports on the component repository, II) Final data users who have access to the stored reports and dashboard through both the Web portal and AI Assistance.
- Interactive Site: This component implements a Web-based interactive site that exposes the system apps and services. The graphical user interface of this component can be parametrized to satisfy different kinds of users, such as policymakers, Data Analysts, and non-expert citizens. Access to these services is given according to a set of predefined user profiles managed by the Security & Access Control unit:

### Data collection plan

**Fig 5:**
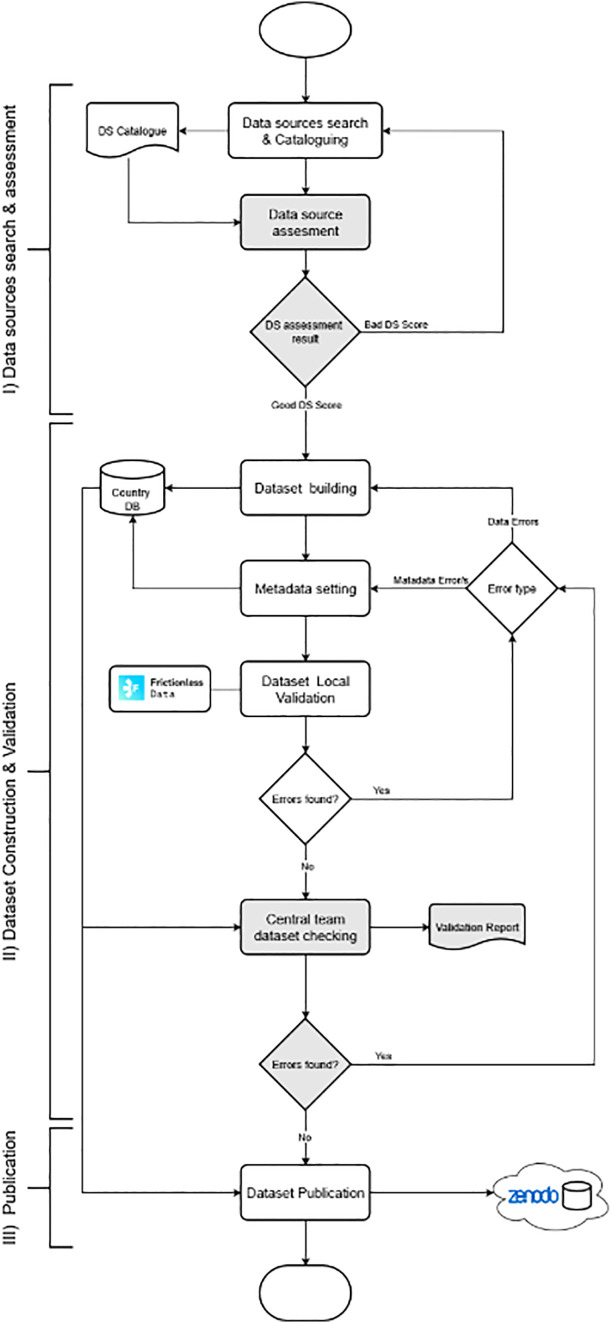
Data collection flowchart: The grey boxes represent the central team tasks, while the white ones indicate the tasks of the partner country teams.

As mentioned in the section “Operationalization of Predictive Variables and Food and Nutrition Security (FNS)”, although the data categories used for FNS monitoring and modeling are defined in the working methodology, the contextual and dependent variables to be used depend on the availability of data in each of the partner countries. There is no consensus in Latin America regarding what socioeconomic, demographic, political, or nutritional data should be collected, and even less so regarding when, where, and how to conduct these studies. The quality, quantity, and formats used vary greatly from one country to another and even within the same region. Therefore, as a first step and before ingesting data into the system, it is necessary to search for and characterize the data sources available in each country (Fig 5).

The data must then be downloaded, cataloged, and integrated consistently. The goal is to obtain a standardized dataset and metadata to feed into the information system. This process is critical and resource-intensive, as these data will be the raw material for model construction and, therefore, for obtaining reliable FNS predictions. This data collection required the formation of interdisciplinary work teams in each partner country, which were supervised and coordinated by the central project team (in Argentina). The process was carried out in three stages: 1) Search and diagnosis of data sources; 2) Construction and validation of datasets; 3) Publication of data in open repositories.

Data source search and assessment: This stage aims to identify relevant data sources in each participating country, including government portals, national surveys, censuses, reports from national and international organizations, and academic publications. The selection of a data source depends on a set of established characteristics: Data must be as recent as possible, dating from after 2010 (prioritizing those data sources that are from the same year as the health variable(s) for each country).

- Include data sources that provide socioeconomic, demographic, and policy information related to health (child-adolescent nutritional status, or mortality in general).
- Include data sources on the health of children and/or adolescents (ages 0–18)
- These sources must be open access, or licenses can be obtained for reuse and publication in open repositories.
- Datasets must guarantee geographic coverage that includes all localities with more than 5000 inhabitants in the country.
- Data with spatial granularity at the city level is prioritized.
- Data must be reliable. Official data sources or those from nationally recognized organizations are prioritized.

The teams in each partner country were responsible for searching and cataloging the relevant data sources in their region. For each source, the following characteristics were documented:

- Source author and reference organization.
- Presence and scope of associated metadata.
- License type/access level of the data.
- Whether the data is covered by personal data protection regulations (if applicable).
- General description of the datasets, including the study population.
- Data date and update period.
- Geographic coverage of the data.
- List of relevant attributes for each dataset.

This documentation, along with data samples, was then used by the core team to assess the availability and quality of socioeconomic, demographic, political, and nutritional information in each country. In cases where the assessment yielded negative results, searches for alternative data sources were conducted, resulting in an iterative search and assessment process.

Dataset Construction and Validation: To construct a single dataset per country, the efforts of this stage focused on the integration and processing of the retrieved data. This processing involved tasks such as data cleaning, normalization, disambiguation, indicator calculations, aggregation/disaggregation (to maintain the same level of granularity across the dataset), among others. The CSV (tabular) format with UTF-8 encoding was chosen to store the resulting data sets. A key aspect of the proposed methodology is the early identification of data problems, that is, before they are incorporated into the system's databases. Indeed, at this stage, special effort was devoted to identifying data errors through a combination of automated and manual validation mechanisms (performed by nutrition experts). Another important aspect of the methodology is the construction of standards-based metadata files that record key information for data traceability. The metadata obtained was organized according to the following details: authors and description of the database, data sources, license for subsequent use, and a brief description of each variable, including a theoretical and empirical definition of each one, its relationship with the health variable, any constrains in its values, and the source and processing methods used to incorporate it into the database.

Furthermore, the validation mechanism to be implemented uses this metadata to automatically detect inconsistencies, omissions, or errors in the data. This metadata definition and validation process was supported by Frictionless Data, a progressive open-source framework for building data infrastructure/data management, data integration, data flows, etc. It includes both data standards and software to work with them. The core component of Frictionless is the Data Package Standard [10]. The Data Package is a comprehensive set of specifications for organizing, documenting, and sharing data in a FAIR-compliant manner. In particular, the Data Package provides a JSON-based scheme for registering metadata. Both Frictionless and Data Package were developed by the Open Knowledge Foundation (OKF), a well-known non-profit organization, with best practice policies on governing openly. In this project, the Data Package-based metadata were recorded with the help of Open Data Editor (ODE), an open-source desktop software also developed by OKF. In addition to supporting metadata definition, ODE implements automatic validation of Frictionless data, identifying the type and location of errors in the dataset. To carry out this metadata definition and validation process, ODE training was conducted for the partner country teams. Once the teams completed constructing the datasets with their metadata, they were validated locally before being sent to the central team.

For each dataset received, the core team performed a more detailed data verification, replicating basic controls and adding controls from the team of nutrition experts. Any issues identified by the core team were reported to the partner countries for addressing, initiating a second iteration of “Dataset Construction and Validation.” This iterative process aims to ensure data quality, a key requirement not only for building reliable open data platforms but also for publishing scholarly articles under an Open Science strategy [11].

### Publication of collected data in open repositories

The publication of the data obtained in the previous steps has two purposes: 1) The publication constitutes an intermediate product of the full data pipeline proposed in the project. Thus, facilitating the traceability and reproducibility of the methodology followed, and providing greater reliability and transparency to the system's output.

2) Besides being the primary input of our system, these data are also valuable for other decision-making processes involving socioeconomic, environmental, health, and policy dimensions. The aim is therefore to make these data accessible and reusable for others in academia, government agencies, and the general public.

In light of these objectives, the FAIR (Findable, Accessible, Interoperable, and Reusable) principles were adopted as criteria for choosing the publication framework [12]. Zenodo has been selected as the preferred publication infrastructure [13]. Zenodo is a free, open-access repository for research outputs funded by the European Commission through the OpenAIRE initiative. In addition to being a FAIR-compliant framework, Zenodo provides long-term data storage along with persistent identifiers (DOIs), ensuring that published items can be permanently cited and located.

Table 1 evidence the total number of variables that will be extracted for each country, grouped according to the dimensions of the theoretical-conceptual approach of the CFEs (political, individual, and environmental dimensions).

**Table 1:** Number of variables to be extracted per country according to the CFEs approach.

	Total number of extracted variables	Variables extracted corresponding to the individual dimension	Variables extracted corresponding to the environmental dimension	Variables extracted corresponding to the political dimension	Health Variables
**Argentina**	126	35	74	12	5
**Chile**	148	28	74	8	38
**Colombia**	144	36	74	16	18
**Paraguay**	111	15	74	3	19
**Peru**	138	24	74	19	21
**Uruguay**	116	23	74	15	4

**Fig 6:**
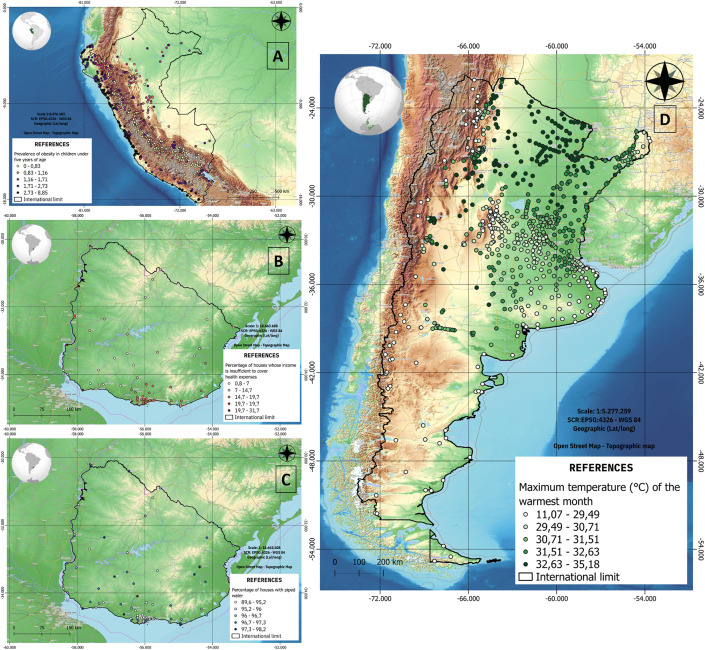
Spatial distribution of health variables and CFEs. Variables in order of appearance: **A)** Prevalence of obesity in children under 5 years (Health variable), **B)** Percentage of houses whose income is insufficient for cover health needs (Political dimension variable), **B)** Percentage of houses with water pipe (Individual dimension variable), and **B)** Maximum temperature of the warmest month (Environmental dimension variable).

Once the databases have been constructed and published, the next step is the statistical and spatial characterization of the CFEs and health variables. As an example, of the expected results Fig 6 shows the preliminary spatial distributions of environmental and health variables in Uruguay, Peru, and Argentina.

### Preliminary Modeling of Health Variables Under Climate Change Scenarios: The Case of Argentina

As supporting Evidence for the Proposed Approach, we present here the modeling of health-related outcomes under projected climate change scenarios in Argentina. Climate change is recognized as one of the defining challenges of the 21st century, with profound adverse effects on human health. However, the application of predictive models to assess specific health outcomes, such as low birth weight (LBW), within the context of climate change remains limited in the literature.

This case study encompasses 657 urban localities across Argentina. For each locality, data on birth weight for live births from the period 2018–2019 were compiled, along with contextual variables characterizing CFEs. These data were also sourced from open-access datasets and remote sensing platforms.

**Fig 7:**
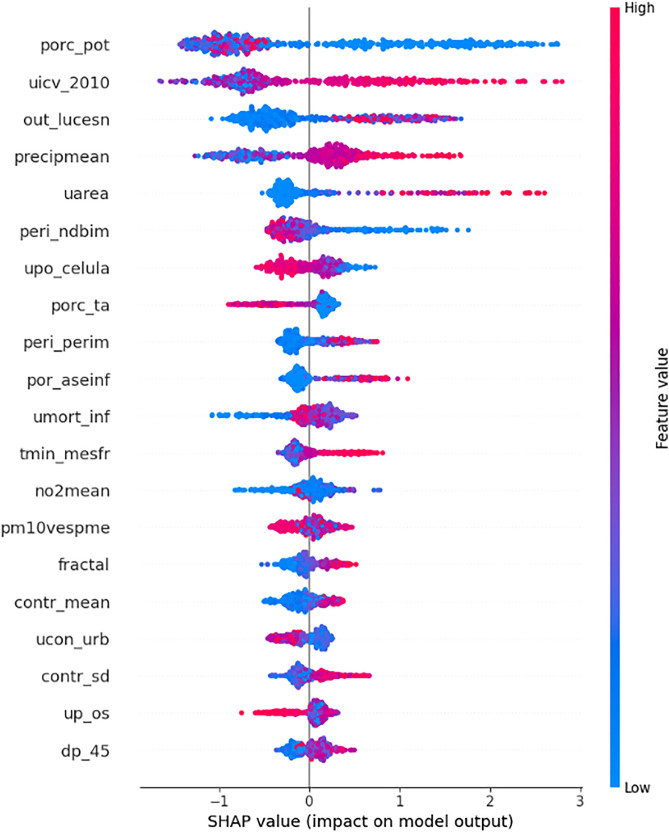
Supporting evidence regarding modelling: Feature importance of the antecedent model for Argentinean localities. Variables in orden of appearence: Percentage of people enrolled in the Potenciar Trabajo plan, quality of life index, and nighttime light radiance in the peri-urban area, mean precipitations, urban area, periurban NDBI, percentage of houses with at least one celphone, percentage of beneficiaries of Tarjeta Alimentar plan, periurban perimeter, percentage of the urban area occupied by informal settlements, infant mortality rate, nitrogen dioxide concentration, particular material concentration (PM10), fractal dimension, texture filter (mean contrast), urban concentration, texture filter (standar deviation contrast), percentage of people with social security coverage, population density (aged 4 to 5 years old).

**Fig 8:**
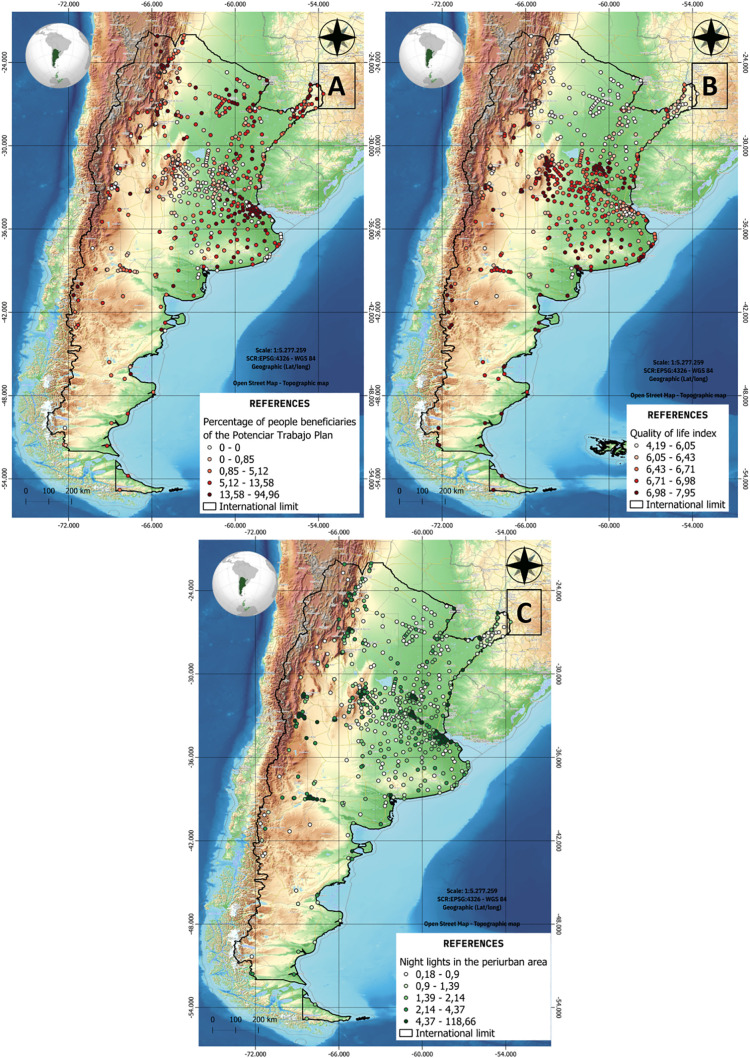
Spatial distribution of the 3 most important variables in the antecedent model. Variables in orden of appearence: **A)** Percentage of people enrolled in the Potenciar Trabajo plan (political dimension), B) quality of life index (political dimension), and C) and nighttime light radiance in the peri-urban area (environmental dimension).

The modeling process was conducted in two stages. First, a comprehensive model was trained using the full set of available predictors. Subsequently, a reduced model was constructed using only the ten most influential variables associated with LBW, as determined by feature importance metrics. Among nine ML algorithms evaluated, the Extreme Gradient Boosting (XGBoost) algorithm demonstrated superior performance and was thus selected for final modeling. Figs 7 and 8 (A, B, and C) present the feature importance of the model created using the Explainer SHAP package (applied by members of the research team previously [14,15]), and the spatial distribution of the third most important variables reported by the training stage for this Argentinean dataset respectively (Percentage of people enrolled in the Potenciar Trabajo plan, quality of life index, and nighttime light radiance in the peri-urban area (as a proxy for urbanization).

To simulate the effects of climate change, annual variation rates were estimated for the top-ranked environmental predictors. Spatial autocorrelation analysis was then performed to evaluate clustering patterns of LBW prevalence. Comparative spatial analyses were conducted between original LBW data and model outputs under simulated conditions. These geospatial analyses were implemented using ArcGIS Pro and QGIS 3.28.

**Fig 9:**
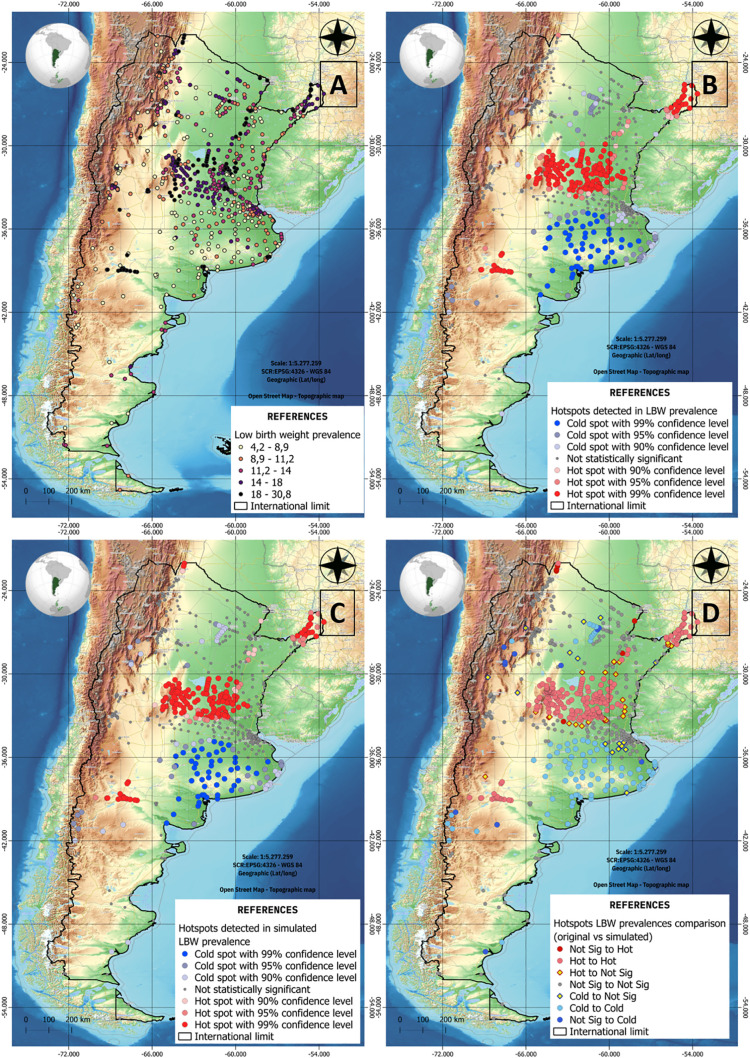
Spatial characterization and patterns detected in the prevalence of original and simulated LBW. In which: **A)** Original LBW prevalence, B) and **C)** Hotspot detection in LBW original and simulated prevalences respectively, and D) hotspot comparison between original and simulated LBW.

Figs 9a-9d illustrate the observed LBW prevalence from previous proposal, as well as the spatial clusters identified under both original and simulated conditions, and their comparison (analyses carried out by project members [16,17]). The final model achieved a coefficient of determination (R²) of 0.88, indicating strong predictive accuracy. Cluster analysis revealed consistent spatial patterns of high LBW prevalence, predominantly concentrated in the central regions of the country. Notably, simulations under projected climate conditions suggest a geographic contraction of these high-prevalence clusters, with intensification toward the country’s center.

These results demonstrate the feasibility of generating data-driven simulations for FNS and CFEs domains using ML approaches, providing an essential foundation for further development within this regional initiative.

### Chat Prototype: Development and Integration of the Virtual Assistant

The proposed ecosystem includes, as a strategic component, a virtual assistant (VA) based on LLM, integrated into the FNS simulation dashboard. The VA will function as an evidence-driven conversational interface, capable of processing natural language queries, invoking relevant predictive models, and presenting results in graphical and textual formats.

The functional architecture adopts a Retrieval Augmented Generation (RAG) approach: the LLM queries a knowledge base derived from the project's ontology and multidimensional data cube. This scheme allows natural language queries to be translated into SPARQL statements, facilitating access to datasets and simulation outputs for non-specialist users, including policymakers and public health teams, without requiring advanced technical skills. The development of the AV was based on the Bloomberg Philanthropies Data for Health initiative, which implemented a prototype for the city of Córdoba, Argentina. In that experience, the interface used a decision tree to guide interaction based on predefined climate and health scenarios. While it was suitable for a controlled demonstration, its architecture showed limitations for dynamic, multinational monitoring of SAN.

Within the framework of the ALSEA project, a transition to an interactive and adaptive system is planned. Domain-specific LLM-based agents and modules are being developed for tasks such as scenario simulation, geospatial query, and policy impact assessment. This incremental process aims to ensure that, upon platform deployment, the AV integrates near-real-time data streams, model outputs, and geospatial analyses, generating contextualized information for each participating country.

**Fig 10:**
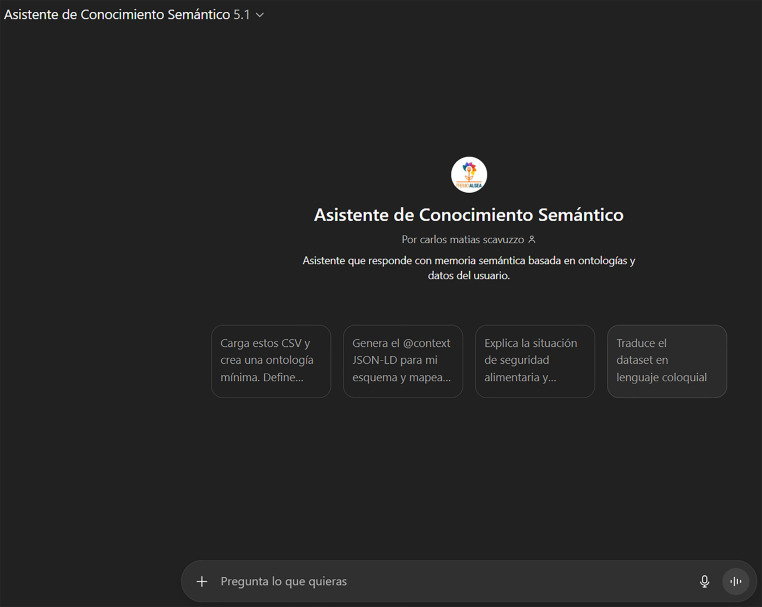
Cover of the LLM agent trained.

The proposed conversational layer aims to bridge the gap between technical analysis and its operational use in public decision support. The AV will be available in a web interface integrated into the dashboard and in mobile applications, facilitating its use by both decision-makers and field teams. In Fig 10, it can be seen the cover of a preliminary agent trained with semantic knowledge linked to FNS and CC.

## Discussion

The ALSEA project deploys a comprehensive architecture that synergistically combines various technological and methodological layers, including multi-resolution satellite observation, predictive modeling using advanced ML techniques, geospatial analysis at different scales, prospective scenario simulation, and decision-support tools. This approach is designed to holistically address FNS in a context marked by climate variability and climate change projections. The proposal responds to the urgent need to integrate, harmonize, and process heterogeneous data sources, such as socioeconomic metrics, environmental indicators, climate variables, and health data, to transform them into high-value strategic inputs for decision-makers and community stakeholders.

The use of satellite information is an advantage for obtaining climate data and other proxies for information that is difficult to access, facilitating decision-making through the use of machine learning platforms and tools. These systems optimize analysis, backed by a team experienced in their use. The advantage of remote sensing data lies in its availability, frequency, and free access.

Also, from a methodological edge, the adoption of a robust ontological framework, combined with a data access system that complies with the FAIR principles, ensures interoperability, traceability, and efficient reuse of information [12]. This semantic infrastructure facilitates comparability between national and regional contexts, allowing the integration of sources as diverse as population surveys, agricultural censuses, and data from optical and radar satellite sensors. At the analytical level, the application of spatial autocorrelation metrics (Moran, LISA), combined with algorithms such as XGBoost and the use of interpretive techniques based on SHAP values, not only reinforces the robustness of the models but also contributes to the transparency and interpretability of the results [18].

The pilot phase implemented in the city of Córdoba, with funding from Bloomberg Philanthropies, provided empirical evidence on the feasibility of integrating interactive interfaces for communicating health and climate scenarios. However, the use of a closed, decision-tree-based structure limited the flexibility to address complex queries and adapt to evolving data. The proposed transition to a virtual assistant based on large-scale language models and a Retrieval Augmented Generation (RAG) approach represents a qualitative leap, enabling open queries, real-time processing, and direct interaction with dynamic models and simulations [19].

In practice, the ALSEA platform has significant potential for strategic planning, evaluation, and adjustment of public policies in FNS. The integration of the interactive dashboard with the virtual assistant allows for intuitive exploration of multi-scale scenarios, identification of spatial and temporal risk patterns, and generation of inputs tailored to the needs of each context. However, its effectiveness will depend on the continuous availability of updated data, the robustness of the local technological infrastructure, and the implementation of training programs that ensure end-users understand and properly use the information.

As described, this is an initial version of a long-term project to develop a policy-making support system. Further functions and capabilities will be added in higher versions. Among others, the following system extensions are considered:

- Ontology-based architecture: An application ontology will be developed to link Indicators, reused data sources, and regional nutrition goals in a consistent knowledge model using an Ontology-Based Data Access (OBDA) approach. At the heart of such an architecture is a knowledge graph, which will be designed to form part of a wider ontology network. Nutrition ontologies, domain-specific controlled vocabularies, and related standards will be evaluated for reuse. Different scales and data granularity will be carefully combined to maintain conceptual robustness in predictions. Through OBDA, databases will be virtually integrated by setting up mappings between dataset attributes and ontology entities. This approach keeps data in their original repositories, while the query-answering services can access the most updated data versions. Such architecture allows both the query endpoints and the AI assistant to be upgraded. Rather than using an SQL interface, a SPARQL endpoint can be deployed as a semantic web service for query answering, employing a high-level vocabulary that is formally defined within the ontology. Regarding LLM-based assistance, this novel, semantic-based approach enables the implementation of a Retrieval-Augmented Generation (RAG) agent that tailors the interaction to the knowledge corpus specified by the aforementioned ontology.
- Validation and application of the project in the field: In the future, it is hoped that the APIs to be developed in WP 3 of this project can be applied in small urban sentinel locations within the project to evaluate the FNS situation using current data. This will also allow for greater accuracy and significance in the models and simulations. Field data collection is a necessary step to complement the open data information.

This proposal is positioned within a broader ecosystem of international initiatives such as the Global Food Security Portal and the FAO and WFP monitoring platforms, providing added value by integrating advanced geospatial analysis capabilities with conversational interfaces powered by artificial intelligence. Its regional and international scalability will depend on the consolidation of strategic alliances, the adoption of open standards, and the strengthening of institutional and community capacities for the management and use of the tool.Threats and risks: The most significant challenges include the need to ensure long-term financial sustainability, prevent technological obsolescence through regular update cycles, and mitigate biases in predictive models associated with incomplete or unrepresentative data. Furthermore, dependence on high-performance digital infrastructure can be a barrier to adoption in territories with low connectivity, requiring differentiated implementation and support strategies.

Thus, the project presents technological and methodological innovation with a solid conceptual foundation and a clearly defined strategic scope. Its success will depend on its validation in real-world environments, its ability to adapt to complex and dynamic scenarios, and its potential to be integrated as a reference tool in food and nutrition security policy planning in response to the challenges of climate change, ensuring not only academic impact but also effective reach to policymakers in nutrition, health, and environmental sectors.
